# Changes in Intestinal Flora and Metabolites in Neonates With Breast Milk Jaundice

**DOI:** 10.3389/fped.2020.00177

**Published:** 2020-05-12

**Authors:** Yaxuan Li, Nan Shen, Jing Li, Rui Hu, Xi Mo, Liqing Xu

**Affiliations:** ^1^Department of Neonatal Intensive Care Unit, Shanghai Children's Medical Center, Shanghai Jiao Tong University School of Medicine, Shanghai, China; ^2^Institute of Pediatric Translational Medicine, Shanghai Children's Medical Center, Shanghai Jiao Tong University School of Medicine, Shanghai, China; ^3^Department of Neonatology, Shanghai First Maternity and Infant Hospital, Shanghai, China

**Keywords:** breast milk jaundice, intestinal flora, intestinal metabolites, *Escherichia*, neonates

## Abstract

**Background:** Breast milk jaundice (BMJ) is the first cause of neonatal jaundice; however, its underlying mechanism is yet to be deciphered. We conducted a study to investigate intestinal flora in neonates with BMJ and used metabolomics to decipher the possible mechanisms by which intestinal flora induces jaundice.

**Methods:** Microbiota collected from the feces of BMJ patients and jaundice-free breastfeeding newborns was used for 16S rRNA sequencing. In addition, differences in fecal metabolites were analyzed using gas chromatography mass spectrometry (GC/MS). The relationship between intestinal microbiota and the differences in fecal metabolites was then analyzed.

**Results:** There was no significant difference in the richness and diversity of intestinal flora between BMJ and the control group; however, there were differences in the structure. At the phylum level, the relative abundance of *Firmicutes* was higher in the control group compared to the BMJ group, whereas *Proteobacteria* was higher in the infants with BMJ. Additionally, at the genus level, the relative abundance of *Haemophilus* was higher in the control group, whereas the relative abundances of *Escherichia, Morganella*, and *Rothia* were lower. More remarkably, the major differences in metabolites between the two groups were glyceric acid, succinic acid, and phenylalanine. Additionally, the abundance of *Escherichia* was positively correlated with succinic acid and cadaverine levels.

**Conclusions:** The intestinal flora colonization status in BMJ patients is immature. This study reports for the first time that the study of intestinal flora, especially *Escherichia*, plays an important role in BMJ, and found that it may be associated with the regulation of succinic acid metabolic pathways.

## Introduction

Neonatal jaundice is one of the most common manifestations observed during the neonatal period. Breast milk jaundice (BMJ) is the first cause of neonatal jaundice, which is characterized by unconjugated hyperbilirubinemia associated with breastfeeding (1). BMJ presents in the first or second week of life, can persist for as long as 12 weeks, and is generally considered to have a good prognosis. However, when the levels of unconjugated bilirubin are very high, it can cross the blood–brain barrier and can cause both short- and long-term neurological dysfunction (2). Interrupting breastfeeding to treat BMJ is controversial and may increase the risk of early termination of breastfeeding (3).

The exact mechanism for BMJ is unknown but may involve decreased caloric intake, which is called breast feeding jaundice, inhibition of hepatic bilirubin excretion, and increased intestinal bilirubin reabsorption. During bilirubin metabolism, gut bacteria have an important role in mediating the transformation of conjugated bilirubin. After excretion from hepatocytes, conjugated bilirubin travels to the small intestine via bile. The intestinal microbiota then converts it to stercobilin for excretion in stool (4). The absence of gut microflora required for the conversion of bilirubin to stercobilin, such as *Clostridium ramosum, Clostridium perfringens, Clostridium difficile*, and *Bacteroides fragilis*, results in higher bilirubin levels in the intestinal tract, which then leads to elevated enterohepatic circulation (5).

Gut bacteria are essential for bilirubin metabolism; however, the role of intestinal microbiome in BMJ development has not been fully deciphered. Intestinal flora metabolizes carbohydrates, proteins, peptides, and digestive enzymes from food and the host. In addition, gut bacteria participate in host metabolism (such as cholesterol, cholic acid, etc.) (6). Hence, metabolic dysfunctions are closely associated with changes in intestinal flora. Intestinal flora and its metabolites affect metabolic homeostasis of host (7).

We investigated the richness, structure, and diversity of intestinal flora in neonates with BMJ and subsequently used metabolomics to understand the possible mechanisms by which intestinal flora induces jaundice. The aim of our study was to understand the role of gut microbiome during early BMJ development, analyze the characteristics of bacterial metabolites, and thereby provide a knowledge base for potential biomarkers for diagnosis of BMJ.

## Methods

### Subjects

All infants in the study were full term and breast-fed and were not administered with antibiotics or probiotics after birth. Mothers were between 20 and 40 years old and were healthy during pregnancy.

The BMJ patients from Shanghai Children's Medical Center and jaundice-free newborns (control group) from Shanghai UIB International Maternity Care Center were selected for the study in January to June 2018. Ten infants with BMJ met the BMJ diagnostic criteria, which included the following: (1) were breastfeed exclusively; (2) jaundice appeared 3–4 days after birth, continued to rise well-beyond the physiological range, and subsided with a delay, and serum bilirubin was mainly unconjugated bilirubin; (3) after the cessation of breast milk for 3 days, jaundice was significantly reduced and bilirubin levels decreased by 50% or greater. (4) Exclusion criteria—pathological jaundice induced by infection, glucose-6-phosphate dehydrogenase deficiency, autoimmune hemolysis, polycythemia, scalp hematoma, intracranial hemorrhage, cholestasis, hypoglycemia, hypothyroidism, hypothermia, and perinatal asphyxia; and (5) infants had proper weight gain. Infants were excluded if weight loss was more than 7% of birth weight, and birth weight did not recover after 7 days, or the average weight gain after 7 days was <30 g/day.

Ten healthy full-term newborns in the control group were exclusively breast fed and had no pathological jaundice, who were matched with the BMJ group in terms of gender, gestational age, and delivery mode.

This study was approved by the Ethics Committee of Shanghai Children's Medical Center affiliated with Shanghai Jiao Tong University School of Medicine. Signed informed consents were obtained from the parents at study enrollment.

### Sample Collection and 16S rRNA Gene Sequencing

Feces were collected from infants soon after admission (within 24 h of admission as far as possible) to avoid the impact of treatment measures on the intestinal flora. Total DNA was extracted by using the QIAamp Fast DNA Stool Mini Kit according to the manufacturer's instructions (Qiagen, Germany). The V3–V4 regions of the 16S rRNA gene were amplified and then sequenced using the Illumina Miseq sequencing platform (Illumina, San Diego, CA). Raw data were filtered to remove adapter sequences and low-quality reads. Clean reads and then paired-end reads with overlaps were merged to tags. Tags were then clustered to operational taxonomic units (OTUs) at 97% sequence similarity. Taxonomic ranks were assigned to OTU representative sequence. Finally, alpha and beta diversity and the different species were analyzed using OTU and taxonomic ranks.

### Metabolomics Analysis

Frozen feces samples (50 mg) from three BMJ neonates and three matched healthy neonates were used for metabolomics analysis. Methanol (800 μl) and internal standard (5 μl; 9.5 mg/ml ribosol) were added to the fecal samples. The samples were then ground and ultrasonically crushed in an ice bath. After centrifugation, the supernatants were transferred to a sample vial and then dried at room temperature. They were then oximated using 20 mg/ml methoxyamino pyridine solution (30 ml) and then derivatized using 30 ml of BSTFA (containing 1% TMCS) for gas chromatography mass spectrometry (GC/MS) analysis.

### Statistical Analysis

SPSS (ver. 22.0) and R software (ver. 3.1.0) were used for statistical analysis of clinical data and infant characteristics. Rarefied alpha diversity and beta diversity indexes were calculated using Quantitative Insights Into Microbial Ecology (QIIME). To determine differences in the microbiome and the significance of the metabolites obtained from GC/MS, comparisons between the groups were performed using Student's *t*-tests or Mann–Whitney test in R (depending on whether the variable was normally distributed). GraphPad Prism software (version 7.00, GraphPad Software, Inc., CA, USA) was used to perform Spearman correlation analysis between the microbiome and the metabolome.

## Results

### Fecal Alpha and Beta Diversity in BMJ and Control Neonates

**Figure 1:**
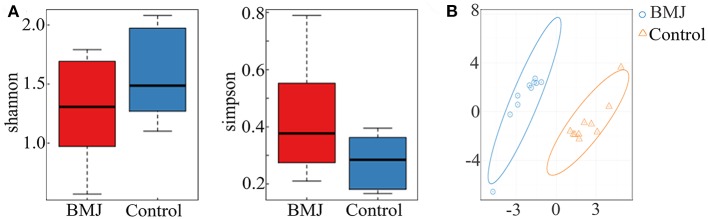
Alpha and beta diversity between the BMJ and control groups. **(A)** The alpha diversity index between BMJ and control groups. **(B)** OTU-based PLS-DA. The scale of horizontal and vertical axes is relative distance, which has no practical significance.

Clinical characteristics of the neonates are depicted in Table 1. There were no significant differences in sex, delivery mode, gestational age, age of admission, and birth weight between the two groups. The peak value of serum total bilirubin in the BMJ group was 18.06 ± 1.33 mg/dl. High-throughput sequencing of 16S rRNA had no significant differences for the alpha diversity index between the BMJ and control groups (Figure 1A). The Shannon index of the BMJ group (1.286 ± 0.413) was lower than that of the control group (1.603 ± 0.365), and the Simpson index (0.412 ± 0.184) was higher than that of the control group (0.273 ± 0.085), but there was no significant difference (*P* = 0.123 and 0.105, respectively).

**Table 1:** Analysis of clinical data.

		**BMJ(*n* = 10)**	**Control (*n* = 10)**	***P* value**
Gender	Male	7	8	1
	Female	3	2	
Delivery	Eutocia	7	7	1
	Cesarean section	3	3	
Gestational age, days, mean±SD		274.00 ± 5.41	272.30 ± 7.62	0.586
Days of sampling, mean ± SD		14.60 ± 5.30	15.80 ± 6.00	0.641
Birth weight, g, mean ± SD		3149.50 ± 304.91	3023.00 ± 249.09	0.323

In order to analyze beta diversity, OTUs were used for partial least squares discrimination analysis (PLS-DA). As shown in Figure 1B, stool samples from the two groups could be roughly separated. Although there was no significant difference in the richness and diversity, the structure of the intestinal flora of BMJ was significantly different from that of healthy children.

### Alteration in Taxa Between the BMJ and Control Groups

**Figure 2:**
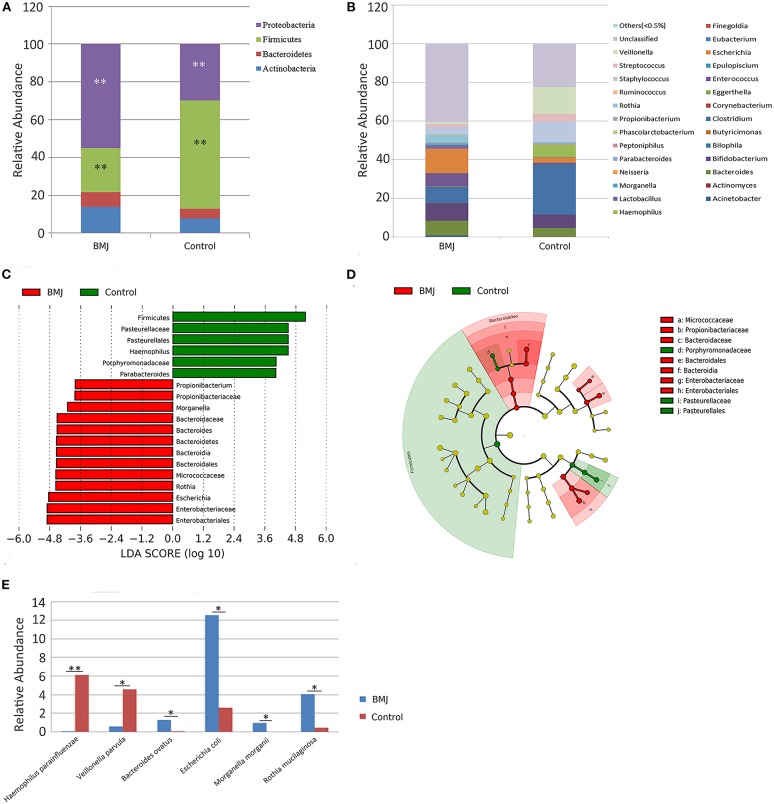
Differences in taxa between the BMJ and control groups. **(A,B)** Differences in relative abundance at the phylum and genus levels between the two groups. **(C,D)** LEfSe analysis shows microbe biomarkers for all taxa in the two groups. **(E)** Differences in relative abundance at the species level between the two groups (*P < 0.05, **P < 0.01).

Considering the changes in the structure of the intestinal flora, the different bacteria were analyzed for all taxa. We particularly measured the differences in the taxa at the phylum and genus levels. At the phylum level, the relative abundance of *Firmicutes* was higher in the control group compared to the BMJ group (*P* = 0.003), whereas *Proteobacteria* was higher in BMJ infants (*P* = 0.009; Figure 2A). Additionally, at the genus level, the relative abundance of *Haemophilus* was higher in the control group (*P* = 0.011), whereas the relative abundance of *Escherichia, Morganella*, and *Rothia* were lower (*P* = 0.029, 0.035, and 0.018, respectively; Figure 2B).

Taxa with linear discriminant analysis (LDA) values > 2.0 are depicted in Figure 2C. The LEfSe cladogram directly shows these important microbe biomarkers for the groups in all taxa (Figure 2D). There were 6 bacteria that were abundant in the fecal samples of the healthy control infants and 13 bacteria that were abundant in the fecal samples from BMJ infants. The relative abundance of *Escherichia* in the BMJ group (12.529 ± 20.781) was higher than that of the control group (2.613 ± 8.053). The data demonstrated that *Escherichia* is the most abundant genus among the different bacteria. Presence of *Escherichia* may be a potential biomarker for BMJ.

We then analyzed the differences in relative abundance at the species level between the two groups. The abundance of *Haemophilus parainfluenzae* and *Veillonella parvula* in the BMJ group was lower compared to that of the control group (*P* = 0.006 and 0.048, respectively), whereas the abundance of *Bacteroides ovatus, Escherichia coli* (*E. coli*), *Morganella morganii*, and *Rothia mucilaginosa* was higher compared to that of the control group (*P* = 0.025, 0.029, 0.035, and 0.018, respectively; Figure 2E).

### Differences in Fecal Metabolites Between the BMJ and Control Groups

**Figure 3:**
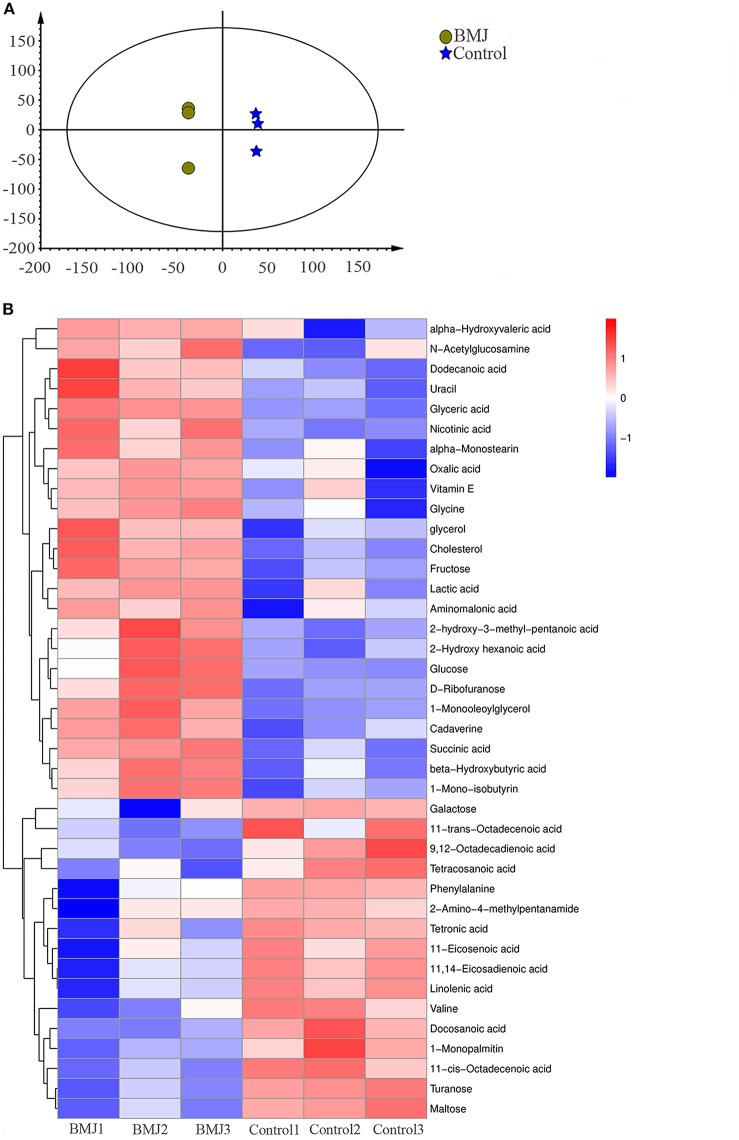
Differences in fecal metabolites between the BMJ and control groups. **(A)** Characteristics of the metabolic patterns in the two groups. The scale of horizontal and vertical axes is relative distance, which has no practical significance. **(B)** Heatmap shows 40 fecal metabolites that were differentially expressed in the two groups.

In addition to the differences in gut microbiome, we studied the differences in metabolite levels induced by the different intestinal flora. The results showed that the metabolic pattern of fecal supernatant samples from BMJ infants had an obvious classification trend with the healthy control group on the multiple statistical score map of Orthogonal Partial Least Square-Discriminant Analysis (OPLS-DA) (Figure 3A).

Compared to healthy breast-fed infants, we identified 40 metabolites with significant differences in BMJ infants (Figure 3B). KEGG (Kyoto Encyclopedia of Genes and Genomes) was used to analyze the metabolic pathways involving these metabolites. We found metabolic abnormalities in fecal supernatants from BMJ infants. The key metabolic changes in BMJ infants were mainly manifested in the pentose phosphate pathway (*P* < 0.0001), amino sugar and nucleotide sugar metabolism (*P* < 0.0001), citrate cycle (*P* = 0.003), alanine, aspartate, and glutamate metabolism (*P* = 0.003), tyrosine metabolism (*P* = 0.003), and other metabolic abnormalities. These pathways were mainly related to glycometabolism, amino acid metabolism, and fatty acid metabolism, indicating that they may play a role in the pathogenesis of BMJ.

### Correlation Between the Differential Fecal Metabolites and Intestinal Microflora

**Figure 4:**
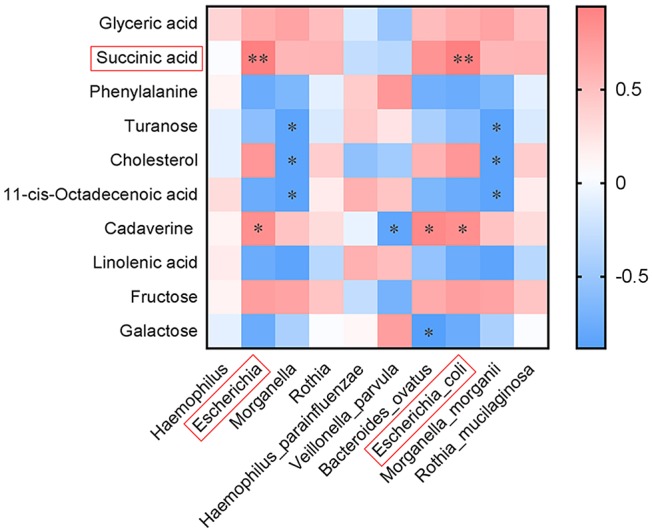
Correlation between the top 10 differential fecal metabolites and intestinal microflora at the genus and species levels (*P < 0.05, **P < 0.01).

Correlation analysis was performed for the differential fecal metabolites and intestinal microflora obtained by high-throughput 16S rRNA sequencing. This was to understand the possible effects of the structural changes of intestinal flora on the metabolic phenotype of BMJ. We analyzed the correlation between the top 10 metabolites and the differential bacteria at the genus and species levels (Figure 4). *Escherichia* or *E. coli* was positively associated with succinic acid (*R* = 0.943, *P* = 0.005) and cadaverine (*R* = 0.829, *P* = 0.042); *Escherichia* or *E. coli* and succinic acid had the most relevant differences.

## Discussion

The reason for the occurrence and development of BMJ is unknown. As an important link in the pathogenesis of neonatal jaundice, enterohepatic circulation has attracted considerable attention. The changes of intestinal flora may be one of the mechanisms that induce BMJ. In this study, we demonstrated, for the first time, that intestinal flora in BMJ infants had unique characteristics and structural changes that affected the metabolic phenotype of BMJ.

16S rRNA sequencing of intestinal flora showed several types of dysbiosis in BMJ infants. At the phylum level, the abundance of *Proteobacteria* in BMJ infants was generally higher. Studies have shown that during the colonization of intestinal flora in the neonatal period, the neonatal meconium had low intestinal flora diversity and was mainly composed of *Proteobacteria* (8). Aerobic bacteria or facultative anaerobes such as *Proteobacteria* began to colonize in the first few hours after birth. Its colonization gradually consumes the oxygen in the intestine, which provides a good condition for the anaerobes such as *Firmicutes* to colonize. The transformation from aerobic bacteria or facultative anaerobes to anaerobes was completed in about 10 days after birth (9, 10). In our study, the imbalance between *Firmicutes* and *Proteobacteria* in BMJ infants indicated that the colonization process of intestinal flora was delayed and was immature until about 2 weeks of age.

The abundance of *Proteobacteria* was generally higher in BMJ infants, but the mechanism is not completely clear. The colonic epithelium is normally hypoxic. However, intestinal inflammation or antibiotic use increases the oxidation in colonic epithelium, which affects the anaerobic state (11, 12). This subsequently induces the increase in *Proteobacteria* numbers through aerobic respiration, resulting in dysbiosis. Hence, changes in intestinal epithelial microenvironment may exist in BMJ neonates.

At the genus level, the abundance of *Escherichia* in BMJ infants was higher. The abundance of *E. coli* at the species level was higher compared to the control group. More remarkably, the average abundance of *Escherichia* was the highest among the different genera between the two groups. Studies have shown that *E. coli* can produce β-glucuronidase (β-GD), which can hydrolyze conjugated bilirubin to unconjugated bilirubin (13). When the abundance of *E. coli* in the intestine is elevated, the production of β-GD increases accordingly, which then increases bilirubin enterohepatic circulation and causes jaundice. However, Miao et al. found that the proportion of *Escherichia* in normal healthy infants was 64.67%, which was significantly higher compared to those observed in BMJ infants (14). The results contrast with what we observed in this study. This may be due to the fact that the average infant age in our study was about 14 days old, which was younger compared to the infants in Miao's study, where the average age was 25 days. The changes of *Escherichia* abundance at different stages of life may have different effects on BMJ.

The colonization of *E. coli* during the neonatal period is a dynamic process. Early colonization of intestinal flora in breast-fed newborns is during the rapid colonization of *E. coli* (15). On approximately the 10th day, the abundance changes to anaerobic bacteria such as *Bifidobacterium* and *Lactobacillus* (16). In our study, however, the abundance of *E. coli* in BMJ neonates is still high around 14 days after birth. This indicates that the colonization status of intestinal flora is immature in BMJ neonates. Hence, early monitoring of fecal *E. coli* may be important for BMJ diagnosis.

Metabolomics could be used to detect and identify various metabolites to accurately reflect the physiological state of organisms. In addition, it can help to identify mechanisms by which the intestinal flora participates in disease and host metabolism (17). In our study, Spearman correlation analysis demonstrated that the amount of *Escherichia* or *E. coli* was positively correlated with two metabolites, succinic acid and cadaverine. Succinic acid is mainly involved in the citrate cycle (18). In humans, the citrate cycle is the most effective way to oxidize glucose or other substances to supply adenosine triphosphate (ATP). ATP is essential for the enzymatic reaction of uridine diphosphate glucuronyl transferase (UDPGT), a rate-limiting enzyme for bilirubin metabolism in the liver (19). We hypothesized that *E. coli* may lead to the development of BMJ by regulating UDPGT activity to affect the citrate cycle. Moreover, succinate plays a key dual regulatory role in inducing pro-inflammatory or anti-inflammatory responses to different intestinal environmental factors (20). The role of succinate in the occurrence and development of BMJ remains to be deciphered.

As a biogenic amine, cadaverine was found to be significantly elevated in BMJ neonates. Polyamines are produced both by the host and by intestinal flora bacteria, and are involved in numerous physiological processes (21). Elevated levels of polyamines may be toxic and are associated with several diseases. Cadaverine levels have also been directly associated with *Escherichia* in Crohn's disease patients, which suggests the presence of intestinal inflammation (22). While their specific roles are largely unknown, they may have a potential role in the occurrence and development of BMJ.

In this study, we selected 10 BMJ neonates after strict selection criteria to determine the changes in intestinal flora and its metabolites to understand the pathogenesis of BMJ. However, there were a few limitations in our study. First, there are still confounders that cannot be completely avoided, especially for mothers, such as maternal diet and maternal antibiotics before pregnancy. Second, our study was a correlation analysis of fecal metabolic profiles. A causal relationship between intestinal flora and jaundice had not yet been established. The specific mechanism needs to be further investigated using blood and other biological samples for a deeper understanding. Besides, this study collected samples at single time points. Longitudinal studies at different stages of BMJ should be performed to comprehensively understand the role of intestinal flora and their metabolites in disease progression. Finally, the sample cohort was small and needs to be expanded to validate our findings. In addition, multiple fecal samples at different stages during neonatal development should be analyzed for a comprehensive understanding of BMJ development.

## Conclusion

Although there were no changes in the richness and diversity of intestinal flora in BMJ neonates, the intestinal flora in BMJ neonates were predominantly colonized with *Escherichia* with an imbalance in *Firmicutes* and *Proteobacteria*. The mechanism of BMJ may be related to the regulation of metabolic pathways by intestinal *Escherichia* by affecting succinic acid, cadaverine, and other metabolite levels. Intestinal flora and its metabolites can be used as biomarkers for diagnosis and severity monitoring of BMJ; furthermore, providing the theoretical and experimental basis of intestinal flora regulation served as one target of BMJ treatment strategies.

## Data Availability Statement

The datasets generated in this study have been deposited to an online repository. This can be accessed at https://www.ncbi.nlm.nih.gov/sra using the accession number PRJNA625544.

## Ethics Statement

The studies involving human participants were reviewed and approved by Ethical Committee of Shanghai Children's Medical Center. Written informed consent to participate in this study was provided by the participants' legal guardian/next of kin.

## Author Contributions

YL collected data and samples, performed analysis, and drafted the initial version of the manuscript. NS carried out data interpretation and reviewed the manuscript. JL had primary responsibility for the study design, data analysis, and interpretation, and reviewed and revised this paper. RH was involved in samples and data collection. XM and LX supervised data collection and were involved in data interpretation.

## Conflict of Interest

The authors declare that the research was conducted in the absence of any commercial or financial relationships that could be construed as a potential conflict of interest.
